# Periprosthetic Joint Infection of Shoulder Arthroplasties: Diagnostic and Treatment Options

**DOI:** 10.1155/2017/4582756

**Published:** 2017-12-20

**Authors:** Bernd Fink, Florian Sevelda

**Affiliations:** ^1^Department of Joint Replacement, General and Rheumatic Orthopaedics, Orthopaedic Clinic Markgröningen gGmbH, Kurt-Lindemann-Weg 10, 71706 Markgröningen, Germany; ^2^Orthopaedic Department, University-Hospital Hamburg-Eppendorf, Martinistrasse 52, 20246 Hamburg, Germany; ^3^Orthopaedic Department, University of Vienna, Vienna, Austria

## Abstract

Periprosthetic joint infection (PJI) is one of the most frequent reasons for painful shoulder arthroplasties and revision surgery of shoulder arthroplasties.* Cutibacterium acnes (Propionibacterium acnes)* is one of the microorganisms that most often causes the infection. However, this slow growing microorganism is difficult to detect. This paper presents an overview of different diagnostic test to detect a periprosthetic shoulder infection. This includes nonspecific diagnostic tests and specific tests (with identifying the responsible microorganism). The aspiration can combine different specific and nonspecific tests. In dry aspiration and suspected joint infection, we recommend a biopsy. Several therapeutic options exist for the treatment of PJI of shoulder arthroplasties. In acute infections, the options include leaving the implant in place with open debridement, septic irrigation with antibacterial fluids like octenidine or polyhexanide solution, and exchange of all removable components. In late infections (more than four weeks after implantation) the therapeutic options are a permanent spacer, single-stage revision, and two-stage revision with a temporary spacer. The functional results are best after single-stage revisions with a success rate similar to two-stage revisions. For single-stage revisions, the microorganism should be known preoperatively so that specific antibiotics can be mixed into the cement for implantation of the new prosthesis and specific systemic antibiotic therapy can be applied to support the surgery.

## 1. Introduction

Periprosthetic joint infection (PJI) of the shoulder joint is a rare but serious complication of shoulder arthroplasties. The mean incidence has been reported to be 1.1%; after reverse arthroplasty, it can be 3.8% and can reach 10% in the subgroup of male, young patient operated on with a reverse prosthesis [1–4]. However, PJI is the most common reason for revisions of shoulder prosthesis made necessary by pain, stiffness, or loosening [5]. Pottinger et al. [6] reported that periprosthetic infections were detected in 56% of 193 shoulder prosthesis revisions. Therefore it is suggested that, until proven otherwise, every report of pain, stiffness, and loosening of the shoulder prosthesis should be regarded as an indication of infection.

Risk factors associated with periprosthetic shoulder infections are posttraumatic osteoarthritis, previous surgery, repeated cortisone injections, systemic corticosteroid treatment and other immunosuppressive medicaments, rheumatoid arthritis, and diabetes mellitus [4, 6, 7]. Richards et al. [5] studied 4,258 patients with shoulder prostheses and found that males were 2.59-times more at risk for infection than females and that reverse total shoulder arthroplasty was associated with a 6.11-higher risk of infection than anatomical shoulder arthroplasty. However, the fact that reverse shoulder arthroplasty is frequently used for revision surgery may cause this difference. Trauma-associated prostheses were associated with a 2.98-greater risk of infection [5].

The microorganisms most commonly associated with periprosthetic infections are the skin pathogens* Staphylococcus *sp. and* Cutibacterium acnes (Propionibacterium acnes)*. Recent studies have shown that the* Cutibacterium acnes (Propionibacterium acnes)* is associated with between 31% and 70% of all periprosthetic shoulder infections and causes many more periprosthetic infections in the shoulder than in other joints, probably because of the proximity of the surgical site to the axillary region [5, 6, 8].

The classification proposed by Tsukayama et al. [31] differentiates between acute early and chronic late infections whereby the threshold between the two is 4 weeks after the surgical intervention. However, other authors regard infections occurring up to 3 months after surgery as early infections [32–36]. Acute periprosthetic infections that arise after many trouble-free years as a result of an infection at a remote site are classified as acute hematogenous infections and are treated in the same way as acute early postoperative infections [31].

PJI of shoulder arthroplasties have different distributions of microorganisms and are less frequent compared to PJI of hip and knee arthroplasties. Clear and standardized concepts for diagnosis and surgical and antibiotic treatment have not been reported in the literature. Because of this inhomogeneity in diagnosis and treatment, the ASES (American Shoulder and Elbow Surgeons) has formed a special committee for the treatment and diagnosis of PJI. This review presents an overview of different diagnostic and therapeutic options and discussion of their advantages and disadvantages.

## 2. Diagnostic Methods

It is not only because of the incidence of infection and the difficulties to detect a slow growing pathogen such as* Cutibacterium acnes* that an accurate, preoperative diagnostics have particular importance in cases of loosened or painful shoulder arthroplasties. These diagnostic tests should be carried out before every revision surgery because evidence for a periprosthetic infection results in a significant change in the treatment. A sufficient preoperative diagnostic may also reduce the amount of unexpected positive cultures in revision shoulder arthroplasty which was 23.9% of 117 revision shoulder arthroplasties in the study of Padegimas et al. [37], of which 57.1% were* Cutibacterium acnes*.

The principles involved in the diagnosis of a periprosthetic infection of the shoulder joint do not differ from those used to investigate hip or knee joints, so much of the experience gained from the more frequently performed hip and knee arthroplasties can be used directly for developing diagnostic tools for assessing infections of shoulder prostheses.

Early infections and acute hematogenous infections are usually associated with local and systemic signs of inflammation. Local signs of inflammation are not always obvious, however, because of the amount of soft tissue covering the shoulder joint. A rapid diagnosis can be achieved by determining the level of C-reactive protein in the blood and the leukocyte count in the joint fluid. In this case, the leukocyte count is usually raised to levels much greater than 10,000/*μ*L [38].

Local and systemic signs of inflammation are absent in cases of late periprosthetic infections, so an accurate diagnosis is much more difficult. In 2011, the Musculoskeletal Infection Society proposed a series of criteria for defining periprosthetic infections; these were adapted in 2014 and proposed that an infection definitely exists when one major criterion or at least three of the five minor criteria are met [39].

The major criteria include

- evidence for organisms with identical phenotype in at least two positive periprosthetic cultures of aspirated joint fluid and/or synovial tissue samples; or
- a fistula communicating with the prosthesis.

The minor criteria include

- elevated erythrocyte sedimentation rate (ESR ≥ 30 mm/h) and level of C-reactive protein (CRP ≥ 10 mg/l) in the serum,
- elevated leukocyte (WBC) count in the joint fluid or positive reaction by leukocyte esterase test strips,
- elevated percentage of neutrophil granulocytes (PMN ≥ 70%) in the joint fluid,
- positive histological assessment of the periprosthetic tissue,
- one single positive culture of periprosthetic tissue or fluid.

The existence of a periprosthetic infection should, in our opinion, always be excluded or proven before a revision arthroplasty is carried out because, on the one hand, a specifically targeted systemic and/or local antibiotic therapy can only be designed on that basis and, on the other hand, the antibiotic therapy can be initiated at the time of surgery. Thus, analyses for PJI should be done preoperatively and should not begin during surgery (e.g., tissue biopsy for bacteriological and histological tests or an intraoperative alpha-defensin test). The intraoperative tests are necessary in our opinion to confirm preoperative diagnosis by obtaining at least two concordant cultures. Some surgeons start the identification of microorganisms intraoperatively and use an empirical broad-spectrum antibiotic treatment [1]. Because the microorganisms most commonly associated with periprosthetic infections are the skin pathogens* Staphylococcus *sp. and* Cutibacterium acnes*, broad-spectrum antibiotics will be sufficient in most cases. However, for resistant* Staphylococcus *sp. and for some Gram-negative microorganisms they are not. In these cases, the initiation of a suitable treatment would not be possible until the microorganism had been detected and identified from samples taken intraoperatively, that is, at a time when leaving bacteria in the periprosthetic tissue had already formed a biofilm around the new implant. In addition, it is useful to obtain an exact differentiation of the pathogen and its resistance pattern so that a systemic antibiotic therapy can be planned preoperatively. This information will also enable the addition of specific antibiotics to the cement used in a one-stage or two-stage revision arthroplasty that are tailored to the pathogen concerned [40, 41]. In this way, local and systemic antibiotic treatments can be devised according to the identity and resistance pattern of the infecting pathogen and so avoid the unnecessary, nonspecific use of broad-spectrum antibiotics with all its disadvantages. In addition, this will also reduce the development of resistance to the antibiotics [37, 38, 40, 41].

We divide the currently available diagnostic methods for demonstrating the presence of a periprosthetic infection or its absence into two groups: direct or specific methods for detecting the pathogen and testing its sensitivity to antibiotics, and indirect or unspecific methods that are unable to provide such information. Indirect, unspecific methods only provide evidence or proof of an infection but leave the questions unanswered of the identity of the pathogen and of its antibiotic susceptibility. Thus, with those considerations in mind, we put great value on the application of specific methods (aspiration or biopsy) of assessment before a revision arthroplasty is carried out.

Imaging methods are nonspecific tests. Early implant loosening or osteolyses (2-3 years after the operation) shown in the radiographies are suspicious for PJI [42]. Scintigraphy is not useful in the first postoperative year because of false positive results due to physiological adaptations processes of the bone to the implant [42]. Moreover, they have a low specificity [42]. Leucocyte-scintigraphy does not have higher sensitivity and specificity, and computed tomography (CT) and magnetic resonance tomography (MRT) do not play any role for diagnosing PJI at the shoulder but may be helpful for visualizing abscess formations and positron emission tomography (PET) in combination with CT is indicated for the latter situation [42].

The CRP value in the blood as a nonspecific test is below 10 mg/L in many cases of periprosthetic infections [42]. Dodson et al. [43] found CRP values higher than 10 mg/L in only 72% of periprosthetic shoulder infections. IL-6 has been shown to be specific but not sensitive for PJI [42]. Thus, it is necessary to use other diagnostics methods in order to prove or exclude the existence of a periprosthetic infection before a revision arthroplasty is carried out.

The aspiration of the joint offers different nonspecific and specific tests. The determination of the cell count in the aspirate is one nonspecific test. Moroder et al. [42] established that a cell count of more than 2000/*μ*l and/or more than 70% of polymorph nuclear leucocytes is indicating a late PJI of the shoulder.

Another nonspecific test is the leucocyte esterase strip test. For diagnosis of PJI of total knee and hip arthroplasties, the sensitivity was between 69% and 81% and the specificity between 93% and 100% [44–46]. However, 17% to 30% of the test was nonreadable because of blood contamination of the aspirate. Centrifugation of the aspirate may improve the readability of the aspirates [47].

A new addition to the range of diagnostic nonspecific tools is the alpha-defensin synovial fluid biomarker assay that has become established as an unspecific diagnostic method in recent years. Sensitivity and specificity of the assay have been reported to be between 97% and 100% [48, 49]. Alpha-defensin is released by leukocytes following contact with bacteria and acts as autogenic antimicrobial agent. It has the advantage that, unlike CRP, systemic inflammatory diseases do not affect it and that previous antibiotic administration does not affect its release or the assay [50, 51]. Frangiamore et al. [52] studied shoulder prostheses and reported a sensitivity of 63% for the test and a specificity of 95%.

One of the specific assays for analysis of the bacteria involves the bacteriological cultivation of preoperative joint aspirates [29, 53–55]. Ince et al. [29] reported a sensitivity of 81.2% in the diagnosis of PJI of the shoulder.

A further direct and specific diagnostic method involves biopsy of periprosthetic tissue. Here, the biopsied material is obtained using biopsy forceps via arthroscopic access. At least 5 samples should be taken for bacteriological cultivation and should be added by additional samples for histological examination or frozen sections. The question of whether the tools between each sample should be changed to avoid contamination is not answered in the literature. However, the utility of this basic precaution seems to be obvious.

It is essential to incubate the synovial fluid and biopsy tissue samples for a sufficiently long period, at least 14 days [39, 40, 56, 57]. This extended incubation time is necessary because, on the one hand, the bacteria causing the periprosthetic infection occur at a very low concentration in the biofilm and, on the other hand, are often sessile; these properties lead to a very low growth rate [56, 58–60]. Especially,* Cutibacterium acnes* (in 31% to 70% of the cases the responsible microorganism for PJI of shoulder arthroplasties) is a very slow growing bacterium and needs a long incubation period for its detection [5, 6, 8]. In our study of 110 PJI of hip and knee, we found that only 27% of these slow growing microorganisms were detected after an incubation time of 7 days and that the remaining 73% first showed bacterial growth during the second week of incubation [40]. Dodson et al. [43] also found that evidence for the presence of bacteria in 11 patients with PJI of the shoulder only appeared during the second week of incubation. Moreover, Pottinger et al. [6] reported an incubation time of up to 28 days for* Cutibacterium acnes *in patients with periprosthetic shoulder infections. Therefore for detection of* Cutibacterium acnes,* cultures need to be held for 14 to 21 days. Using the bloodstream infection samples and the automatic detection of culture, the delay is now less than 14 days for almost all the pathogens except few, like* Mycobacteria*.

The synovial tissue can also be analyzed using PCR methods to detect the microorganism. The advantage of PCR is that the result is available after few hours and PCR technique can now detect most antibiotic resistances. A disadvantage is the quite high percentage of false positive results due to the detection of not only living bacteria [56, 61].

The advantage of biopsy is the possibility of combining the different diagnostic methods of cultivation and histological examination on several tissue samples [39, 62, 63]. Dilisio et al. [64] studied 41 shoulder arthroplasties and found that biopsy is more reliable than aspiration of the synovial fluid and could accurately confirm or rule out the presence of an infection. The biopsy method was associated with a sensitivity of 100%, a specificity of 100%, a positive predictive value of 100%, and a negative predictive value of 100%, whereas the aspiration method was found to have a sensitivity of only 16.7%, a specificity of 100%, a positive predictive value of 100%, and a negative predictive value of 58.3%. Therefore, we suggest synovial biopsy in cases where the other indirect and direct diagnostic methods did not lead to a clear decision on periprosthetic infection and could not identify the microorganism.

## 3. Treatment of Early Infections

The treatment of acute postoperative and hematogenous periprosthetic infections involves a radical surgical debridement of the periprosthetic tissue and a radical synovectomy. This is then followed by a thorough irrigation (also with antiseptic fluids) of the tissue. These are usually open procedures, with the prosthesis inlay being exchanged at the same time. Arthroscopic irrigation does not allow such a radical approach and is associated with lower rates of success than those attained with open debridement and inlay exchange, as seen in the publications of Choi et al. [65] and Byren et al. [66]. Because the onset of infection is often unknown with precision in hematogenous periprosthetic infections, the success rate is lower than in acute postoperative infections [67].

The bacterium causing these infections is mostly unknown at the time of surgery and initiation of the antibiotic therapy. Therefore an empirical antibiotic treatment has to be started until the microorganism is identified and the specific antibiotic therapy can be adapted to the susceptibility of the microorganism. Zimmerli et al. [36] and Trampuz and Zimmerli [68] give great importance to the use of rifampicin for retaining the prosthesis because it is active against nonresistant bacteria in the biofilm. For infected hip and knee arthroplasties, Zimmerli et al. [36] achieved a success rate of 100% in the treatment of 12 periprosthetic infections using a combination of ciprofloxacin and rifampicin; only 58% success was achieved when ciprofloxacin was combined with a placebo for the treatment of a similar number of patients. Berdal et al. [33] reported 82% success with an antibiotic combination of rifampicin and ciprofloxacin for treating 29 patients. An explanation for this success was suggested to be the ability of rifampicin to affect sensitive, sessile, Gram-positive pathogens in the bacterial biofilm [36, 69, 70]. Fluoroquinolones such as ciprofloxacin are effective against Gram-negative bacteria in the early biofilm [69, 71–73]. Thus, Aboltins et al. [32] were successful in treating 15 of 17 postoperative early Gram-negative infections with ciprofloxacin (nine cases of a mixed infection with staphylococci were treated in combination with rifampicin) while Martínez-Pastor et al. [34] noted that treatment with fluoroquinolones was a positive factor in the treatment of 47 patients with Gram-negative infections. In our own study of infected knee and hip arthroplasties, we chose vancomycin as the combination partner for rifampicin for the first days until the microorganism has been identified because a high level of resistance to fluoroquinolones such as ciprofloxacin exists in our own population and in other centres too [67, 74–76]. Aboltins et al. [32] decided on a combination of vancomycin and other antibiotics administered over a mean period of five weeks as the initial intravenous therapy in 9 of 17 cases with mixed Gram-negative and Gram-positive infections. In our own study of infected knee and hip arthroplasties, we achieved a success rate of 82% when treating acute infections in the first days with a combination of rifampicin and vancomycin followed by a specific antibiotic treatment for a whole period of six weeks [67].

There is little or no published information about how long the antibiotic therapy should actually last. While Zimmerli et al. [70] recommend three months for infections of hip endoprostheses and six months for infected knee prostheses, most authors favour continuing antibiotic therapy until the inflammation parameters have normalised. Several factors led to our decision to carry out a standardized therapy of 6 weeks. Firstly, there is no evidence that a prolonged antibiotic treatment has a positive effect on retention of the prosthesis. Secondly, a prolonged antibiotic therapy is more likely to lead to a masking of the infection and a delay in identifying a treatment failure than to prevent it [67]. In our own experience, an early recognition of a treatment failure leads to an earlier revision of the infected prosthesis. Thirdly, the level of resistance to the antibiotic is increased when treatment failure occurs after a prolonged antibiotic administration [77].

## 4. Treatment of Late Infections

Procedures that can be considered for the treatment of late periprosthetic infections include antibiotic administration alone, debridement of the soft tissue, sine-sine resection arthroplasty, a permanent spacer, and one-stage or two-stage septic revision. Treatment with antibiotics alone is not really an option because the bacteria in the biofilm cannot be eliminated in this way. This was the reason for Coste et al. [10] observing a reinfection rate of 60%. Simple removal of the infected prosthesis and conversion to a sine-sine resection arthroplasty resulted in an improved reinfection rate of 30% according to Coste et al. [10] and even of 0% as reported by Romanò et al. [17]. However, joint function following sine-sine resection arthroplasty is considered to be poor [12, 17] (Table 1).

## 5. Permanent Spacer

The implantation of a spacer after removal of the infected prosthesis results in a very much better joint functionality. Some authors leave the implanted spacer permanently in position and achieve reproducibly low levels of reinfection, even down to 0%, and a satisfactory joint function (Table 2). The spacer acts as a depot for an antibiotic and releases it into the infected prosthesis bed whereby the local concentration of the antibiotic active substance is very much higher than that achievable by systemic administration of the drug. It is also possible to prepare a tailor-made antibiotic/cement mixture, based on the specific resistance and sensitivity pattern of the pathogen concerned. The spacer also maintains the correct tension in the soft tissues and preserves the length of the arm, which in turn leads to better functionality (Tables 1 and 2).

## 6. Two-Stage Revision

**Figure 1:**
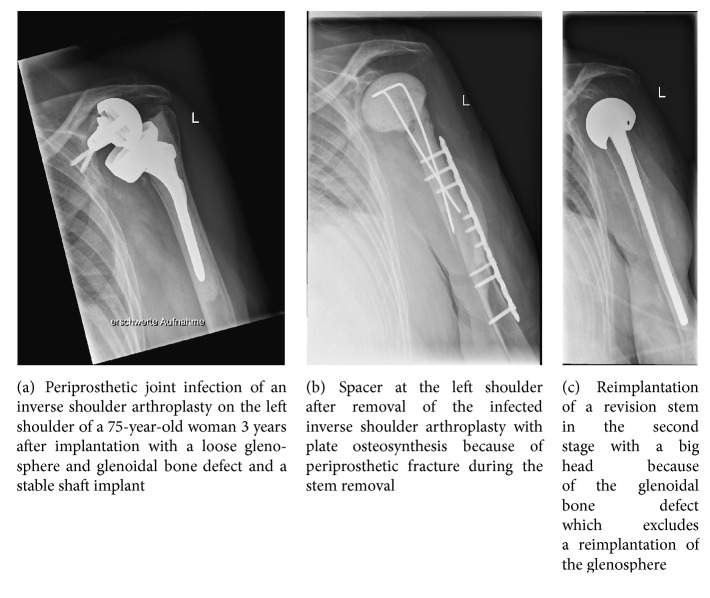


Two-stage revision surgery is the most common method for treating infected prostheses (Figures 1(a)–1(c)). A general advantage of the two-stage concept is that surgical debridement is carried out twice, whereby the second operation enables the eradication of residual organisms remaining after the initial debridement. Since the cement of a spacer is not used for permanent fixation of an implant, the mechanical quality of the cement is not of primary importance and a higher proportion of antibiotic can be added to the cement. It has been possible to achieve a survival rate using two-stage revision concepts for infected shoulder arthroplasties of between 60% and, most commonly, 100% (Table 3). By reducing contractures, the reimplantation of a prosthesis during a two-stage revision procedure is technically easier than after a sine-sine resection arthroplasty (Table 3). Since the rotator cuff is often insufficient following debridement, it is recommended that a reverse shoulder prosthesis be reimplanted. Using this concept, Li et al. [28] achieved a median Constant score of 53.

Most studies use the same antibiotic mixed into the cement of the spacer or provided in the industrially preformed spacer [78]. Some authors use vancomycin and tobramycin as local antibiotics on a regular basis because they have a broad spectrum of activity [79]. However, not all bacteria can be successfully treated with these agents (e.g., some Gram-negative organisms), so this is an argument for investigating the antibiotic resistance pattern of the isolated bacteria and selecting a specific antibiotic for the treatment.

An alternative procedure involves antibiotic-releasing beads. A disadvantage of this method is that it is only possible to use industrially prepared beads and they only contain gentamicin or vancomycin. Moreover, arm shortening and instability occur and mobilization becomes very difficult. This in turn usually makes reimplantation of a prosthesis much more difficult because of scarring, tissue contraction, and disuse osteoporosis. In addition, particles of zirconium dioxide abraded during mobilization could lead to third-body-wear damage to the reimplanted prosthesis.

## 7. One-Stage Revision

**Figure 2:**
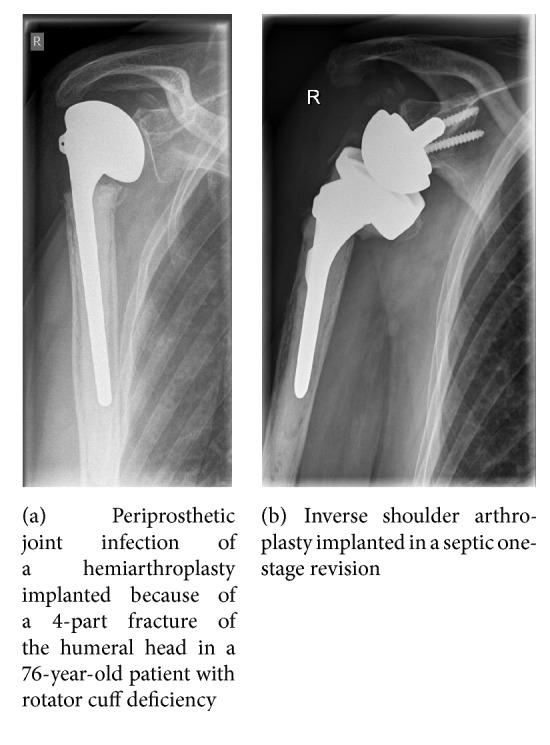


The advantage of the one-stage revision is that only one operation is required (Figures 2(a) and 2(b)). On the other hand, functional problems with a sine-sine resection arthroplasty and associated arm shortening and instability, as well as potential spacer fracture, abraded cement particles from the spacer, or bone resorption resulting from the presence of the spacer, can be avoided. In most cases, antibiotic-impregnated cement is used for the reimplantation whereby the antibiotic that is added to the cement or is already contained in it is specific for the pathogen concerned [29, 30]. Even though the preoperative identifying of the pathogen in aspirated synovial fluid or tissue biopsy is not fully satisfactory, for one-stage procedure it is helpful to know the pathogens and their susceptibility to antibiotics. Only then can a specific antibiotic mixture be added to the bone cement and enable a local antibiotic therapy [29, 30]. Recent studies using this concept have achieved infection-free survival of between 90% and 100% (Table 4).

The functional outcomes of one-stage revisions depend on the integrity of the rotator cuff following debridement and the type of prosthesis used (Table 4). Ince et al. [29] achieved a Constant score of 33.6 but only implanted one reverse shoulder prosthesis in a cohort of 16 patients. Klatte et al. [30] showed that the reverse shoulder prosthesis, with a Constant score of 61, was very much better than the bipolar head prosthesis with a Constant score of 56 or a hemiarthroplasty with a Constant score of 43. A study of one-stage revision by Beekman et al. [1] provided support for these data with a Constant score of 55.6%.

Nelson et al. [80] and Cuff et al. [23] did not observe any difference in the level of eradication observed after one-stage and two-stage revisions. George et al. [81] undertook a systematic search of relevant publications and found significantly better clinical outcomes after one-stage revisions (mean Constant score of 51) than after two-stage revisions (mean Constant score of 44). In the same report, treatments involving a permanent spacer achieved a mean Constant score of 31 and the sine-sine resection arthroplasty a mean Constant score of 32. The rates of eradication of infection were similar for all four procedures (86.7% for the sine-sine resection arthroplasty, 94.7% for the one-stage revision, 90.8% for the two-stage revision, and 95.6% for the permanent spacer). These results support the concept of the one-stage revision if the pathogen has been characterized.

## Figures and Tables

**Table 1:** Resection arthroplasty.

Authors	*N*	Follow-up (years)	Systemic antibiotic treatment	Freedom from infection (%)	Score
Braman et al. 2006 [9]	7	1.7		100	
Coste et al. 2004 [10]	10	2.8	No information	70	30 CS
Rispoli et al. 2007 [11]	13	8.3	No information	100	
Sperling et al. 2001 [12]	21			71.4	
Debeer et al. 2006 [13]	7	0.9			26 CS
Verhelst et al. 2011 [14]	11	1.9			46 CS
Ghijselings et al. 2013 [15]	6	2.1			28 CS
Weber et al. 2011 [16]	5	4		100	33 CS
Romanò et al. 2012 [17]	6	3.5		100	32 CS

**Table 2:** Permanent spacer.

Authors	*N*	Follow-up (years)	Systemic antibiotic treatment	Local antibiotic treatment	Freedom from infection (%)	Score
Coffey et al. 2010 [18]	4	1.8		Gentamicin	100	57 CS
Coste et al. 2004 [10]	3	2.8	No information	No information	100	38 CS
Jerosch and Schneppenheim 2003 [19]	2				100	
Themistocleous et al. 2007 [20]	4				100	
Stine et al. 2010 [21]	15	2.4			100	50 DASH
Ghijselings et al. 2013 [15]	4	3.3				21 CS
Romanò et al. 2012 [17]	15	3			93.3	34 CS
Mahure et al. 2016 [22]	9	4			100	57 ASES

**Table 3:** Two-stage revision.

Authors	*N*	Follow-up (years)	Systemic antibiotic treatment	Local antibiotic treatment	Freedom from infection (%)	Score
Coffey et al. 2010 [18]	12	1.8		Gentamicin	100	57 CS
Coste et al. 2004 [10]	10	2.8	No information	No information	60	35 CS
Cuff et al. 2008 [23]	10				100	
Jerosch and Schneppenheim 2003 [19]	8				100	
Mileti et al. 2004 [24]	4	7.4			100	
Seitz Jr. and Damacen 2002 [25]	5	4.8			100	
Sperling et al. 2001 [12]	3				100	
Stine et al. 2010 [21]	12	2.4			100	
Strickland et al. 2008 [26]	19				63.2	
Weber et al. 2011 [16]	4	4			100	40 CS
Romanò et al. 2012 [17]	17	3.8			100	38 CS
Buchalter et al. 2017 [27]	19	5.25			78	69 ASES
Li et al. 2016 [28]	8	1.65			100	53 CS

**Table 4:** One-stage revision.

Authors	*N*	Follow-up (Years)	Systemic antibiotic treatment	Local antibiotic treatment	Freedom from infection (%)	Score
Coste et al. 2004 [10]	3	2.8	No information	No information	100	66 CS
Cuff et al. 2008 [23]	7				100	
Ince et al. 2005 [29]	16	5.7			100	33,6 CS
Sperling et al. 2001 [12]	2				50	
Beekman et al. 2010 [1]	11	0.9			90,9	51 CS
Klatte et al. 2013 [30]	35	2.7			94	51 CS

## References

[1] Beekman P. D. A., Katusic D., Berghs B. M., Karelse A., De Wilde L. (2010). One-stage revision for patients with a chronically infected reverse total shoulder replacement. *The Journal of Bone & Joint Surgery (British Volume)*.

[2] Hudek R., Gohlke F. (2013). Endoprosthesis infections of the shoulder: Diagnosis and therapy algorithm. *Der Orthopäde*.

[3] Padegimas E. M., Maltenfort M., Ramsey M. L., Williams G. R., Parvizi J., Namdari S. (2015). Periprosthetic shoulder infection in the United States: Incidence and economic burden. *Journal of Shoulder and Elbow Surgery*.

[4] Singh J. A., Sperling J. W., Schleck C., Harmsen W. S., Cofield R. H. (2012). Periprosthetic infections after total shoulder arthroplasty: A 33-year perspective. *Journal of Shoulder and Elbow Surgery*.

[5] Richards J., Inacio M. C. S., Beckett M. (2014). Patient and procedure-specific risk factors for deep infection after primary shoulder arthroplasty. *Clinical Orthopaedics and Related Research*.

[6] Pottinger P., Butler-Wu S., Neradilek M. B. (2012). Prognostic factors for bacterial cultures positive for Propionibacterium acnes and other organisms in a large series of revision shoulder arthroplasties performed for stiffness, pain, or loosening. *The Journal of Bone & Joint Surgery*.

[7] Wirth M. A., Rockwood C. A. (1994). Complications of shoulder arthroplasty. *Clinical Orthopaedics and Related Research*.

[8] Piper K. E., Jacobson M. J., Cofield R. H. (2009). Microbiologic diagnosis of prosthetic shoulder infection by use of implant sonication. *Journal of Clinical Microbiology*.

[9] Braman J. P., Sprague M., Bishop J., Lo I. K., Lee E. W., Flatow E. L. (2006). The outcome of resection shoulder arthroplasty for recalcitrant shoulder infections. *Journal of Shoulder and Elbow Surgery*.

[10] Coste J. S., Reig S., Trojani C., Berg M., Walch G., Boileau P. (2004). The management of infection in arthroplasty of the shoulder. *The Journal of Bone & Joint Surgery (British Volume)*.

[11] Rispoli D. M., Sperling J. W., Athwal G. S., Schleck C. D., Cofield R. H. (2007). Pain relief and functional results after resection arthroplasty of the shoulder. *The Journal of Bone & Joint Surgery (British Volume)*.

[12] Sperling J. W., Kozak T. K. W., Hanssen A. D., Cofield R. H. (2001). Infection after shoulder arthroplasty. *Clinical Orthopaedics and Related Research*.

[13] Debeer P., Plasschaert H., Stuyck J. (2006). Resection arthroplasty of the infected shoulder: A salvage procedure for the elderly patient. *Acta Orthopædica Belgica*.

[14] Verhelst L., Stuyck J., Bellemans J., Debeer P. (2011). Resection arthroplasty of the shoulder as a salvage procedure for deep shoulder infection: Does the use of a cement spacer improve outcome?. *Journal of Shoulder and Elbow Surgery*.

[15] Ghijselings S., Stuyck J., Debeer Prof. P. (2013). Surgical treatment algorithm for infected shoulder arthroplasty A retrospective analysis of 17 cases. *Acta Orthopædica Belgica*.

[16] Weber P., Utzschneider S., Sadoghi P., Andress H.-J., Jansson V., Müller P. E. (2011). Management of the infected shoulder prosthesis: A retrospective analysis and review of the literature. *International Orthopaedics*.

[17] Romanò C. L., Borens O., Monti L., Meani E., Stuyck J. (2012). What treatment for periprosthetic shoulder infection? Results from a multicentre retrospective series. *International Orthopaedics*.

[18] Coffey M. J., Ely E. E., Crosby L. A. (2010). Treatment of glenohumeral sepsis with a commercially produced antibiotic-impregnated cement spacer. *Journal of Shoulder and Elbow Surgery*.

[19] Jerosch J., Schneppenheim M. (2003). Management of infected shoulder replacement. *Archives of Orthopaedic and Trauma Surgery*.

[20] Themistocleous G., Zalavras C., Stine I., Zachos V., Itamura J. (2007). Prolonged implantation of an antibiotic cement spacer for management of shoulder sepsis in compromised patients. *Journal of Shoulder and Elbow Surgery*.

[21] Stine I. A., Lee B., Zalavras C. G., Hatch G., Itamura J. M. (2010). Management of chronic shoulder infections utilizing a fixed articulating antibiotic-loaded spacer. *Journal of Shoulder and Elbow Surgery*.

[22] Mahure S. A., Mollon B., Yu S., Kwon Y. W., Zuckerman J. D. (2016). Definitive treatment of infected shoulder arthroplasty with a cement spacer. *Orthopedics*.

[23] Cuff D. J., Virani N. A., Levy J. (2008). The treatment of deep shoulder infection and glenohumeral instability with debridement, reverse shoulder arthroplasty and post-operative antibiotics. *The Journal of Bone & Joint Surgery (British Volume)*.

[24] Mileti J., Sperling J. W., Cofield R. H. (2004). Reimplantation of a shoulder arthroplasty after a previous infected arthroplasty. *Journal of Shoulder and Elbow Surgery*.

[25] Seitz W. H., Damacen H. (2002). Staged exchange arthroplasty for shoulder sepsis. *The Journal of Arthroplasty*.

[26] Strickland J. P., Sperling J. W., Cofield R. H. (2008). The results of two-stage re-implantation for infected shoulder replacement. *The Journal of Bone & Joint Surgery (British Volume)*.

[27] Buchalter D. B., Mahure S. A., Mollon B., Yu S., Kwon Y. W., Zuckerman J. D. (2017). Two-stage revision for infected shoulder arthroplasty. *Journal of Shoulder and Elbow Surgery*.

[28] Li F. L., Jiang C. Y., Lu Y., Zhu Y. M., Li X. (2016). Efficacy analysis of two-stage reverse total shoulder arthroplasty for treating postoperative deep infection after surgeries for proximal humeral fractures. *Beijing Da Xue Xue Bao*.

[29] Ince A., Seemann K., Frommelt L., Katzer A., Loehr J. F. (2005). One-stage exchange shoulder arthroplasty for peri-prosthetic infection. *The Journal of Bone & Joint Surgery (British Volume)*.

[30] Klatte T. O., Junghans K., Al-Khateeb H. (2013). Single-stage revision for peri-prosthetic shoulder infection: outcomes and results. *The Bone & Joint Journal*.

[31] Tsukayama D. T., Estrada R., Gustilo R. B. (1996). Infection after total hip arthroplasty: a study of the treatment of one hundred and six infections. *The Journal of Bone & Joint Surgery*.

[32] Aboltins C. A., Dowsey M. M., Buising K. L. (2011). Gram-negative prosthetic joint infection treated with debridement, prosthesis retention and antibiotic regimens including a fluoroquinolone. *Clinical Microbiology and Infection*.

[33] Berdal J.-E., Skråmm I., Mowinckel P., Gulbrandsen P., Bjørnholt J. V. (2005). Use of rifampicin and ciprofloxacin combination therapy after surgical debridement in the treatment of early manifestation prosthetic joint infections. *Clinical Microbiology and Infection*.

[34] Martínez-Pastor J. C., Muñoz-Mahamud E., Vilchez F. (2009). Outcome of acute prosthetic joint infections due to gram-negative bacilli treated with open debridement and retention of the prosthesis. *Antimicrobial Agents and Chemotherapy*.

[35] Soriano A., García S., Bori G. (2006). Treatment of acute post-surgical infection of joint arthroplasty. *Clinical Microbiology and Infection*.

[36] Zimmerli W., Widmer A. F., Blatter M., Frei R., Ochsner P. E. (1998). Role of rifampin for treatment of orthopedic implant-related staphylococcal infections. *Journal of the American Medical Association*.

[37] Padegimas E. M., Lawrence C., Narzikul A. C. (2017). Future surgery after revision shoulder arthroplasty: the impact of unexpected positive cultures. *Journal of Shoulder and Elbow Surgery*.

[38] Fink B., Lass R. (2016). Diagnostic Algorithm for Failure Analysis of Painful Total Hip Arthroplasties. *Zeitschrift für Orthopädie und Unfallchirurgie*.

[39] Parvizi J., Gehrke T. (2014). Definition of periprosthetic joint infection. *The Journal of Arthroplasty*.

[40] Fink B., Makowiak C., Fuerst M., Berger I., Schäfer P., Frommelt L. (2008). The value of synovial biopsy, joint aspiration and C-reactive protein in the diagnosis of late peri-prosthetic infection of total knee replacements. *The Journal of Bone & Joint Surgery (British Volume)*.

[41] Schäfer P., Fink B., Sandow D., Margull A., Berger I., Frommelt L. (2008). Prolonged bacterial culture to identify late periprosthetic joint infection: a promising strategy. *Clinical Infectious Diseases*.

[42] Moroder P., Gerhardt C., Renz N., Trampuz A., Scheibel M. (2016). Diagnostik und Management des Endoprotheseninfekts am Schultergelenk. *Obere Extremität*.

[43] Dodson C. C., Craig E. V., Cordasco F. A. (2010). Propionibacterium acnes infection after shoulder arthroplasty: A diagnostic challenge. *Journal of Shoulder and Elbow Surgery*.

[44] Deirmengian C., Kardos K., Kilmartin P. (2015). The Alpha-defensin Test for Periprosthetic Joint Infection Outperforms the Leukocyte Esterase Test Strip. *Clinical Orthopaedics and Related Research*.

[45] Parvizi J., Jacovides C., Antoci V., Ghanem E. (2011). Diagnosis of periprosthetic joint infection: The utility of a simple yet unappreciated enzyme. *The Journal of Bone & Joint Surgery*.

[46] Shafafy R., McClatchie W., Chettiar K. (2015). Use of leucocyte esterase reagent strips in the diagnosis or exclusion of prosthetic joint infection. *The Bone & Joint Journal*.

[47] Ruangsomboon P., Chinprasertsuk S., Khejonnit V., Chareancholvanich K. (2017). Effect of Depth of Centrifuged Synovial Fluid on Leukocyte Esterase Test for Periprosthetic Joint Infection. *Journal of Orthopaedic Research*.

[48] Deirmengian C., Kardos K., Kilmartin P., Cameron A., Schiller K., Parvizi J. (2014). Diagnosing Periprosthetic Joint Infection: Has the Era of the Biomarker Arrived?. *Clinical Orthopaedics and Related Research*.

[49] Deirmengian C., Kardos K., Kilmartin P., Cameron A., Schiller K., Parvizi J. (2014). Combined measurement of synovial fluid a-defensin and C-reactive protein levels: Highly accurate for diagnosing periprosthetic joint infection. *Journal of Bone and Joint Surgery - American Volume*.

[50] Deirmengian C., Kardos K., Kilmartin P., Gulati S., Citrano P., Booth R. E. (2015). The Alpha-defensin Test for Periprosthetic Joint Infection Responds to a Wide Spectrum of Organisms. *Clinical Orthopaedics and Related Research*.

[51] Shahi A., Parvizi J., Kazarian G. S. (2016). The Alpha-defensin Test for Periprosthetic Joint Infections Is Not Affected by Prior Antibiotic Administration. *Clinical Orthopaedics and Related Research*.

[52] Frangiamore S. J., Saleh A., Grosso M. J. (2015). *α*-Defensin as a predictor of periprosthetic shoulder infection. *Journal of Shoulder and Elbow Surgery*.

[53] Barrack R. L., Jennings R. W., Wolfe M. W., Bertot A. J. (1997). The Value of Preoperative Aspiration Before Total Knee Revision. *Clinical Orthopaedics and Related Research*.

[54] Duff G. P., Lachiewicz P. F., Kelley S. S. (1996). Aspiration of the knee joint before revision arthroplasty. *Clinical Orthopaedics and Related Research*.

[55] Mont M. A., Waldman B. J., Hungerford D. S. (2000). Evaluation of preoperative cultures before second-stage reimplantation of a total knee prosthesis complicated by infection. A Comparison-Group Study. *The Journal of Bone & Joint Surgery*.

[56] Gollwitzer H., Diehl P., Gerdesmeyer L., Mittelmeier W. (2006). Diagnostic strategies in cases of suspected periprosthetic infection of the knee: A review of the literature and current recommendations. *Der Orthopäde*.

[57] Ince A., Rupp J., Frommelt L., Katzer A., Gille J., Löhr J. F. (2004). Is ‘aseptic’ loosening of the prosthetic cup after total hip replacement due to nonculturable bacterial pathogens in patients with low-grade infection?. *Clinical Infectious Diseases*.

[58] Costerton J. W. (2005). Biofilm theory can guide the treatment of device-related orthopaedic infections. *Clinical Orthopaedics and Related Research*.

[59] Gallo J., Kolar M., Novotny R., Rihakova P., Ticha V. (2003). Pathogenesis of prosthesis-related infection. *Biomedical Papers*.

[60] Neut D., Van Horn J. R., Van Kooten T. G., Van Der Mei H. C., Busscher H. J. (2003). Detection of Biomaterial-Associated Infections in Orthopaedic Joint Implants. *Clinical Orthopaedics and Related Research*.

[61] Holmes S., Pena Diaz A. M., Athwal G. S., Faber K. J., O'Gorman D. B. (2017). Neer Award 2017: A rapid method for detecting Propionibacterium acnes in surgical biopsy specimens from the shoulder. *Journal of Shoulder and Elbow Surgery*.

[62] Atkins B. L., Athanasou N., Deeks J. J. (1998). Prospective evaluation of criteria for microbiological diagnosis of prosthetic-joint infection at revision arthroplasty. *The OSIRIS Collaborative Study Group*.

[63] Pandey R., Drakoulakis E., Athanasou N. A. (1999). An assessment of the histological criteria used to diagnose infection in hip revision arthroplasty tissues. *Journal of Clinical Pathology*.

[64] Dilisio M. F., Miller L. R., Warner J. J. P., Higgins L. D. (2014). Arthroscopic tissue culture for the evaluation of periprosthetic shoulder infection. *Journal of Bone and Joint Surgery - American Volume*.

[65] Choi H.-R., Von Knoch F., Zurakowski D., Nelson S. B., Malchau H. (2011). Can implant retention be recommended for treatment of infected TKA?. *Clinical Orthopaedics and Related Research*.

[66] Byren I., Bejon P., Atkins B. L. (2013). One hundred and twelve infected arthroplasties treated with 'DAIR' (debridement, antibiotics and implant retention): antibiotic duration and outcome. *Journal of Antimicrobial Chemotherapy*.

[67] Fink B., Schuster P., Schwenninger C., Frommelt L., Oremek D. (2017). A Standardized Regimen for the Treatment of Acute Postoperative Infections and Acute Hematogenous Infections Associated With Hip and Knee Arthroplasties. *The Journal of Arthroplasty*.

[68] Trampuz A., Zimmerli W. (2005). New strategies for the treatment of infections associated with prosthetic joints. *Current Opinion in Investigational Drugs*.

[69] Widmer A. F., Wiestner A., Frei R., Zimmerli W. (1991). Killing of nongrowing and adherent Escherichia coli determines drug efficacy in device-related infections. *Antimicrobial Agents and Chemotherapy*.

[70] Zimmerli W., Trampuz A., Ochsner P. E. (2004). Prosthetic-joint infections. *The New England Journal of Medicine*.

[71] Abdi-Ali A., Mohammadi-Mehr M., Agha Alaei Y. (2006). Bactericidal activity of various antibiotics against biofilm-producing Pseudomonas aeruginosa. *International Journal of Antimicrobial Agents*.

[72] Renz N., Perka C., Trampuz A. (2016). Management of periprosthetic infections of the knee. *Der Orthopäde*.

[73] Yassien M., Khardori N., Ahmedy A., Toama M. (1995). Modulation of biofilms of Pseudomonas aeruginosa by quinolones. *Antimicrobial Agents and Chemotherapy*.

[74] Diekema D. J., Pfaller M. A., Schmitz F. J. (2001). Survey of infections due to Staphylococcus species: frequency of occurrence and antimicrobial susceptibility of isolates collected in the United States, Canada, Latin America, Europe, and the Western Pacific region for the SENTRY Antimicrobial Surveillance Program, 1997–1999. *Clinical Infectious Diseases*.

[75] Nimmo G. R., Bell J. M., Mitchell D., Gosbell I. B., Pearman J. W., Turnidge J. D. (2003). Antimicrobial resistance in Staphylococcus aureus in Australian teaching hospitals, 1989-1999. *Microbial Drug Resistance*.

[76] Zervos M. J., Hershberger E., Nicolau D. P. (2003). Relationship between fluoroquinolone use and changes in susceptibility to fluoroquinolones of selected pathogens in 10 United States teaching hospitals, 1991–2000. *Clinical Infectious Diseases*.

[77] Chastre J., Luyt C.-E., Combes A., Trouillet J.-L. (2006). Use of quantitative cultures and reduced duration of antibiotic regimens for patients with ventilator-associated pneumonia to decrease resistance in the intensive care unit. *Clinical Infectious Diseases*.

[78] Magnan B., Bondi M., Vecchini E., Samaila E., Maluta T., Dall'Oca C. (2014). A preformed antibiotic-loaded spacer for treatment for septic arthritis of the shoulder. *Musculoskeletal Surgery*.

[79] Haddad S., Corona P. S., Reverté M. M., Amat C., Flores X. (2013). Antibiotic-impregnated cement spacer as a definitive treatment for post-arthroscopy shoulder destructive osteomyelitis: Case report and review of literature. *Strategies in Trauma and Limb Reconstruction*.

[80] Nelson G. N., Davis D. E., Namdari S. (2016). Outcomes in the treatment of periprosthetic joint infection after shoulder arthroplasty: A systematic review. *Journal of Shoulder and Elbow Surgery*.

[81] George D. A., Volpin A., Scarponi S., Haddad F. S., Romanò C. L. (2016). Does exchange arthroplasty of an infected shoulder prosthesis provide better eradication rate and better functional outcome, compared to a permanent spacer or resection arthroplasty? a systematic review Orthopedics and biomechanics. *BMC Musculoskeletal Disorders*.

